# *Cryptosporidium* infection in rural Gambian children: Epidemiology and risk factors

**DOI:** 10.1371/journal.pntd.0007607

**Published:** 2019-07-26

**Authors:** M. Jahangir Hossain, Debasish Saha, Martin Antonio, Dilruba Nasrin, William C. Blackwelder, Usman N. Ikumapayi, Grant A. Mackenzie, Mitchell Adeyemi, Momodou Jasseh, Richard A. Adegbola, Anna W. Roose, Karen L. Kotloff, Myron M. Levine

**Affiliations:** 1 Medical Research Council Unit The Gambia at the London School of Hygiene & Tropical Medicine (LSHTM), London School of Hygiene & Tropical Medicine, Banjul, The Gambia; 2 Medicine and Clinical, GlaxoSmithKline Vaccines, Wavre, Belgium; 3 Center for Vaccine Development and Global Health, University of Maryland School of Medicine, Baltimore, MD, USA; 4 Institute for Global Health, University of Maryland, Baltimore, MD, United States of America; 5 GM Murdoch Children’s Research Institute, Melbourne, Australia; University of North Carolina at Chapel Hill, UNITED STATES

## Abstract

**Background:**

*Cryptosporidium* is a major pathogen associated with diarrheal disease in young children. We studied *Cryptosporidium* diarrhea in children enrolled in the Global Enteric Multicenter Study (GEMS) in rural Gambia.

**Methods:**

We recruited children <5 years of age with moderate-to-severe diarrhea (MSD) for 3 years (2008–2010), and children with either MSD or less severe diarrhea (LSD) for one year (November 2011-November 2012) at sentinel health centers. One or more randomly selected controls were matched to each case. Stool samples were tested to identify *Cryptosporidium* by immunoassay. A subset of randomly selected case-controls pairs were tested for *Cryptosporidium* species. We investigated the epidemiology of, and evaluated possible risk factors for, *Cryptosporidium-*positive diarrhea.

**Results:**

We enrolled 1938 cases (1381 MSD, 557 LSD) and 2969 matched controls; 231/1929 (12.0%) of diarrhea cases and 141/2962 (4.8%) of controls were positive for *Cryptosporidium*. Most *Cryptosporidium* diarrhea cases (85.7%, 198/231) were aged 6–23 months, and most (81.4%, 188/231) occurred during the rainy season. *Cryptosporidium hominis (C*. *hominis)* was the predominant (82.6%) species. We found associations between increased risk of *Cryptosporidium*-positive MSD or LSD, or both, with consumption of stored drinking water and certain animals living in the compound—cow, cat (MSD only) and rodents (LSD only). Larger households, fowl living in the compound, and the presence of *Giardia* infection were associated with decreased risk of *Cryptosporidium* MSD and LSD.

**Conclusion:**

*Cryptosporidium*-positive diarrhea is prevalent in this setting, especially at 6–23 months of age. The preponderance of *Cryptosporidium* infection in the rainy season and increased risk of *Cryptosporidium*-positive diarrhea with consumption of stored drinking water suggest water-borne transmission. Further investigation is needed to clarify the role of animals and contamination of stored drinking water in *Cryptosporidium* transmission.

## Author summary

*Cryptosporidium*, a protozoan parasite, is one of the most common diarrheal pathogens in young children living in developing countries. We describe the prevalence and risk factors for *Cryptosporidium* diarrhea in under-five children in The Gambia using data from the Global Enteric Study (GEMS), conducted in seven developing countries in Asia and Africa (2008–2012). We enrolled 1938 diarrhea cases and 2969 matched controls. We found that 12.0% of diarrhea cases and 4.8% controls were positive for *Cryptosporidium*. Most (85.7%) *Cryptosporidium* diarrhea cases were aged 6–23 months, and most (81.4%) occurred during the rainy season. *Cryptosporidium hominis* was the predominant species (82.6%). We found that consumption of stored drinking water and animals (cow, cat, rodents) living in the compound are potential risk factors for *Cryptosporidium* diarrhea. Improved drinking water storage may reduce the burden of *Cryptosporidium* diarrhea in a resource poor hygienic and sanitation setting.

## Introduction

Diarrhea is the second leading cause of morbidity and mortality in children less than 5 years old, causing approximately 600,000 annual deaths, mostly in developing countries [1]. *Cryptosporidium*, a protozoan parasite, is transmitted like other diarrheal pathogens by the fecal-oral route and is more prevalent in patients with HIV/AIDS, in whom it causes severe and prolonged gastrointestinal illness [2–5]. However, the recent Global Enteric Multicenter Study (GEMS) found that *Cryptosporidium* was the third most common pathogen contributing to moderate-to-severe diarrhea (MSD) in children aged less than 5 years, irrespective of HIV prevalence [6, 7]. A multisite birth cohort community-based study (MAL-ED) also detected a high burden of *Cryptosporidium* associated mild and severe infectious diarrhea among children aged 0–24 months [8, 9]. In addition to the substantial burden of gastroenteritis, *Cryptosporidium* with or without concomitant diarrhea has been associated with growth faltering and weight loss [10–13], and with lowered physical fitness, decreased cognitive function [14] and increased mortality [2, 6, 7]. *Cryptosporidium* is associated with an estimated 48,000 global deaths per year in children aged less than five years [12].

In sub-Saharan Africa, the prevalence of, and risk factors for *Cryptosporidium* infection have been studied mostly through cross-sectional surveys over limited time periods with relatively few *Cryptosporidium*-positive cases [15–18]. The MAL-ED study documented risk factors of *Cryptosporidium* only in diarrhea cases of any severity and the effect of co-infection with other enteric pathogens were not assessed [19]. In contrast with the global burden and severity of diarrheal disease with *Cryptosporidium*, information on transmission in developing countries is limited [20, 21]. GEMS was a comprehensive study conducted for several years and using standardised diagnostic methods to determine the disease burden, epidemiology and risk factors for a wide variety of pathogens that may cause diarrhea (23–26). The GEMS study detected that *Cryptosporidium* was among the leading etiologies of MSD in eastern Gambia, accounting for 12% and 8% of MSD in 0–11 months and 12–23 months, respectively [6]. In this paper we report secondary analyses of GEMS data in The Gambia in order to characterize the epidemiology of *Cryptosporidium* infection in diarrhea cases and their controls and to identify risk factors for *Cryptosporidium*-associated diarrhea.

## Methods

### Ethics statement

The study was approved by The Gambia Government/MRC joint Ethics Committee (SCC # 1054) and IRB of University of Maryland, Baltimore (HM-HP-00040030). Written informed consent was obtained from parents or guardians of study participants.

### Study population and study subjects

#### Study area

The Gambia GEMS site was located in Basse, a rural area of 1,111 km^2^ in the Upper River Region (URR) of eastern Gambia with a population included in Basse Health and Demographic Surveillance System (BHDSS). The population was enumerated at household visits every four months with records of births, deaths, vaccination, marriage, and migration in and out of the surveillance area. HIV prevalence is low (<2%) in this setting [22], but malaria is endemic. Houses usually have walls and floors of mud or clay bricks. Roofs are usually thatched of long grass or made of corrugated tin. The average number of household members is about 25, and many families live together in the same courtyard. Most of the households (97%) have a traditional pit latrine. The households usually have domestic animals, including chickens, goats, cattle, donkeys, and horses. Most of the inhabitants (75%) are subsistence farmers producing maize and groundnuts; and a much smaller proportion are involved in small-trade business. The mean per capita income in The Gambia is US$ 440 per annum [23]. There are two distinct seasons, the dry season from November to April, and the wet and rainy season from May to October.

#### Cases

The case definition, selection and enrollment criteria have been described elsewhere [6, 24]. In summary, children aged 0–59 months with diarrhea who resided in the BHDSS area and sought care at any of six sentinel health centres were eligible for screening. Diarrhea was defined as passage of 3 or more loose or watery stools (as perceived by the mother or caretaker) in a 24-hour period [25]. Enrollment was confined to children with an acute diarrheal episode, defined by onset of illness within the last 7 days and no diarrhea for at least 7 days before the onset of the current episode. For the initial 3 years (December 2007-December 2010), only children with MSD were enrolled. MSD was defined as diarrhea with the presence of at least one of the following criteria: sunken eyes, reduced skin turgor, visible or reported blood in the stool, administration or prescription of intravenous fluid, or recommendation of hospitalization. In the final year (November 2011-November 2012), children with either MSD or less severe diarrhea (LSD) were eligible for enrollment. LSD was defined as diarrhea that did not meet any of the criteria for MSD. Eligible children were enrolled after obtaining consent and adequate stool (at least 3 gm).

#### Controls

For each case, one or more randomly selected controls were enrolled. Controls were residents of the BHDSS population, had no diarrhea in the last seven days, and were matched by age (± 2 months for cases aged 0–11 and ± 4 months for cases aged 12–59 months), sex and community. Controls were enrolled within 2 weeks of the corresponding case’s enrollment.

#### Stool sample collection and laboratory testing

Both cases and controls provided a stool sample at the time of enrollment. GEMS used a standardized protocol for stool collection, transport and processing [24, 26]. Samples were tested for a wide range of pathogens, including *Cryptosporidium* [24, 26]. A commercial immunoassay (Tech Lab, Inc, Blacksburg, VA, USA) was used for *Cryptosporidium* testing. Samples from a randomly selected subset of case-control pairs, as well as 10 subjects who died, were tested to by TaqMan Array Card (TAC)-based real-time polymerase chain reaction (PCR) [27].

#### *Cryptosporidium* diarrhea cases

**Fig 1:**
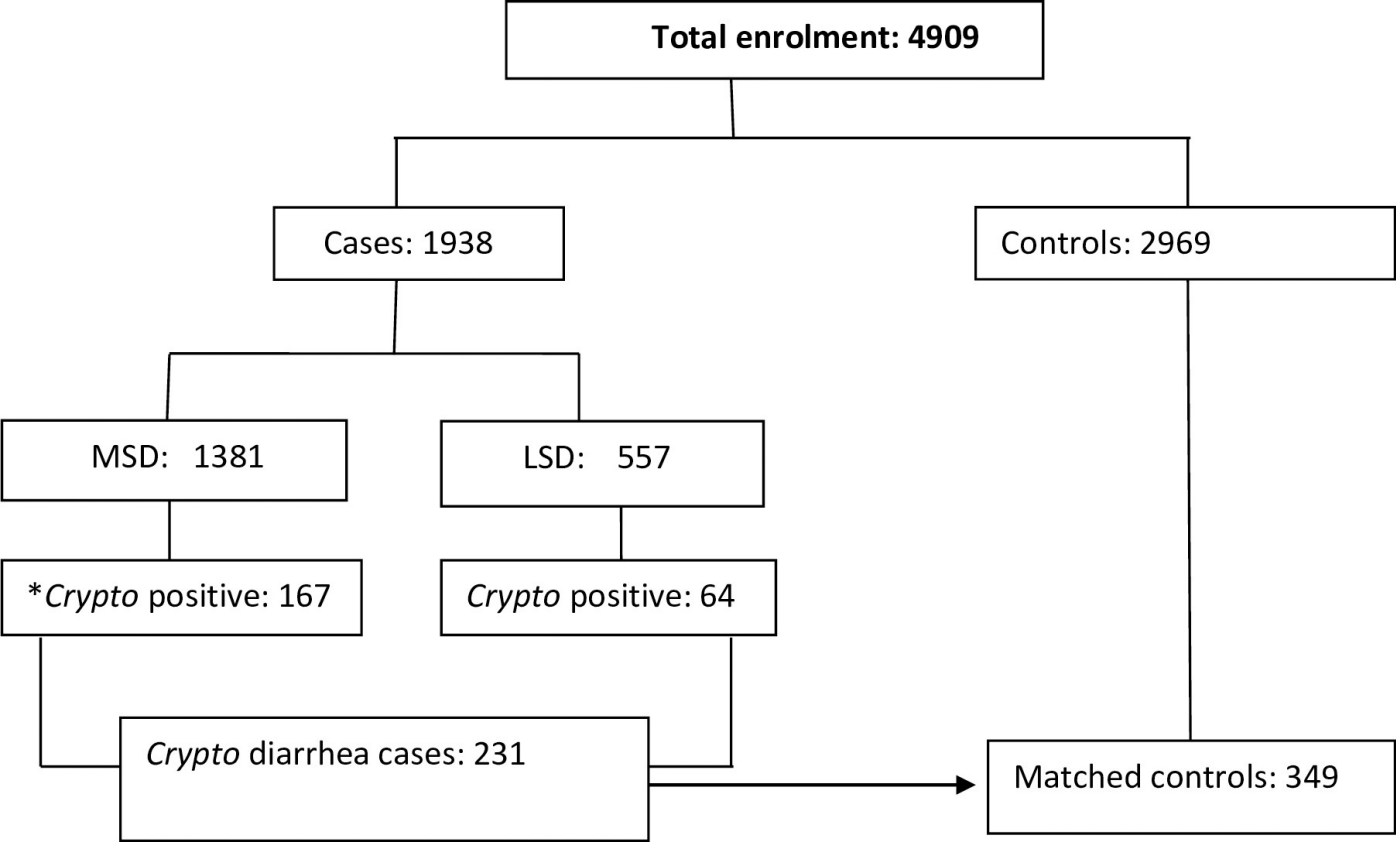
Flow chart of enrollment of *Cryptosporidium* diarrhea cases and their matched controls. *Crypto -Cryptosporidium MSD = Moderate-to-severe diarrhea LSD = Less Severe Diarrhea.

MSD or LSD cases positive for *Cryptosporidium* by EIA were designated as *Cryptosporidium* diarrheal cases. *Cryptosporidium* diarrheal cases and their matched controls were used to investigate risk factors for *Cryptosporidium*-associated diarrhea (Fig 1).

#### Data collection

Data collection in GEMS has been described elsewhere [6, 24]. In brief, we collected information on socio-demographic, drinking water sources, sanitation and hygienic facilities and the presence of animals in the compound from parents or caretakers during enrollment of cases at Sentinel Health Centres (SHCs) and controls at home. Anthropometric measurements including weight and height or recumbent length were taken using standardized procedures [24].

### Data management and analysis

Analysis was done using SAS version 9.4 (SAS Institute, Cary, NC, USA), Stata SE 12.0 (StataCorp. 2011, College Station, Texas, USA), R 3.5.1 [28] and Microsoft Excel. Results with p < 0.05 were generally considered statistically significant; we used p < 0.10 in evaluating differences between associations with Cryptosporidium-positive MSD and LSD in multivariable modeling (i.e., in evaluating interaction terms).

Proportions were measured for categorical data. Differences in proportions were assessed by chi-square test. Continuous data were described by means (SD) or medians (range). Data on building materials and household possessions (e.g., electricity, television, radio, phone, bicycle, car, boat, refrigerator and finished floor and number of sleeping rooms in the dwellings) were used to construct a wealth index [24, 29] as a measure of socio-economic status.

Length or height was determined as the median of three repeated measurements. A height-for-age Z-score (HAZ) was calculated according to WHO guidelines (31), with HAZ < -2 used to define low height-for-age or stunting. Extreme HAZ values of (<-6 or >+6) were excluded from analysis.

The attributable fraction in the exposed (AFE) was calculated for the age range (0–23 months) for which *Cryptosporidium* was a significant pathogen for the entire GEMS study. From the population attributable fractions and total estimated cases with *Cryptosporidium* identified at the Gambia site [6, 30], the number of cases attributable to *Cryptosporidium* was estimated for the age groups 0–11 months and 12–23 months, separately for MSD and LSD. AFE is the ratio of the number of cases attributable to *Cryptosporidium* to the total cases positive for *Cryptosporidium*.

Associations of *Cryptosporidium* with diarrhea overall and within strata of age, sex, season, and type of diarrhea (MSD or LSD) were evaluated using conditional logistic regression.

Analysis of associations with *Cryptosporidium*-positive diarrhea was restricted to *Cryptosporidium*-positive cases and their matched controls. First we fit univariate conditional logistic regression models for any *Cryptosporidium*-positive diarrhea with single factors as covariates. Starting with variables associated with *Cryptosporidium*-positive diarrhea with a p ≤ 0.2 threshold, we fit multivariable conditional logistic regression models using a backward elimination stepwise process to identify a set of factors, each associated with p < 0.05. We then developed separate models for MSD and LSD by including the interaction of each factor with an indicator variable for MSD or LSD. We could not include a “main effect” for MSD/LSD because cases and controls were matched on MSD/LSD. We evaluated associations with socio-demographic variables, breastfeeding status, water source and hygiene variables, animals living in the compound, HAZ score, and presence of other potential pathogens (rotavirus, *Shigella*, norovirus, adenovirus 40/41, ETEC-ST, *Giardia*, *Entamoeba histolytica*, and *Ascaris lumbricoides*).

## Results

### Descriptive Epidemiology of *Cryptosporidium* infection in cases and controls

We enrolled 1938 cases (1381 MSD and 557 LSD) and 2969 matched controls (Fig 1). Of the study participants tested for *Cryptosporidium*, 231 of 1929 cases (12.0%) and 141 of 2962 controls (4.8%) were positive (p = <0.001); data for *Cryptosporidium* were missing for 9 cases and 7 controls.

Table 1 shows the prevalence of *Cryptosporidium* in cases and controls within categories of age, sex, season, and type of diarrhea (MSD or LSD). Prevalence was higher in cases than in controls for both MSD (12.2% vs. 4.8%, p<0.001) and LSD (11.5% vs. 4.6%, p<0.001). *Cryptosporidium* prevalence was similar in children with MSD (167/1381, 12.1%) and LSD (64/557, 11.5%) (p = 0.71 by z-test for proportions).

**Table 1:** Prevalence of *Cryptosporidium* in diarrhea cases and their matched controls by age, sex, season of enrollment and type of diarrhea.

	Diarrhea cases (N = 1929)	Matched controls (N = 2962)		
	*Cryptosporidium* positive/ Total^a^ n/N (%)	*Cryptosporidium* positive/Total^a^ n/N (%)	Odds ratio (95% CI)^b^	p^b^
**Age (months)**^**c**^				
0–5	14/160 (8.8)	9/209 (4.3)	2.0 (0.8–4.8)	0.120
6–11	99/580 (17.1)	54/833 (6.5)	3.7 (2.4–5.6)	<0.001
12–17	56/452 (12.4)	35/636 (5.5)	2.5 (1.6–4.2)	<0.001
18–23	43/359 (12.0)	23/531 (4.3)	3.0 (1.7–5.2)	<0.001
24–59	19/378 (5.0)	20/753 (2.7)	2.0 (1.0–3.8)	0.051
**Sex**^**c**^				
Male	131/1043 (12.6)	63/1588 (4.0)	3.9 (2.7–5.6)	<0.001
Female	100/886 (11.3)	78/1374 (5.7)	2.1 (1.5–2.9)	<0.001
**Season**^**c**^					
Dry (Nov-April)	43/920 (4.7)	30/1467 (2.0)	2.7 (1.6–4.3)	<0.001
Wet (May-Oct)	188/1009 (18.6)	111/1495 (7.4)	2.9 (2.2–3.9)	<0.001
**Type of diarrhea**				
MSD	167/1372 (12.2)	105/2180 (4.8)	2.9 (2.2–3.9)	<0.001
LSD	64/557 (11.5)	36/782 (4.6)	2.7 (1.7–4.2)	<0.001

^a^ Total shown is the number with a non-missing result for presence of *Cryptosporidium*; the result was missing for 9 cases and 7 controls.

^b^ From conditional logistic regression using the Firth penalized likelihood.

^c^ Controls are classified in the same age group, sex and season as the corresponding case. The selection criteria for controls resulted in some controls who were 6 months of age when the case was 5 months of age, and vice versa. Similar differences between case and control age occurred for ages 17 months and 18 months. Season was also different for cases and controls in some cases.

### Proportion of *Cryptosporidium*-positive cases attributable to *Cryptosporidium*

AFE was estimated as 76% and 70% for MSD at ages 0–11 months and 12–23 months, respectively. The corresponding estimates for LSD were 65% and 64%. For the age range 0–23 months, AFE was 73% for MSD and 65% for LSD.

### Prevalence of *Cryptosporidium* infection by age and sex

*Cryptosporidium* prevalence was higher in cases than in controls within all age groups and for both males and females (Table 1). Prevalence in cases was much higher in children aged 6–23 months than in younger infants or children aged 24–59 months, and similar between males and females. Odds ratios for *Cryptosporidium* prevalence in cases versus controls were also highest at ages 6–23 months. Prevalence in cases was similar for males and females.

### Prevalence of *Cryptosporidium* by season

**Fig 2:**
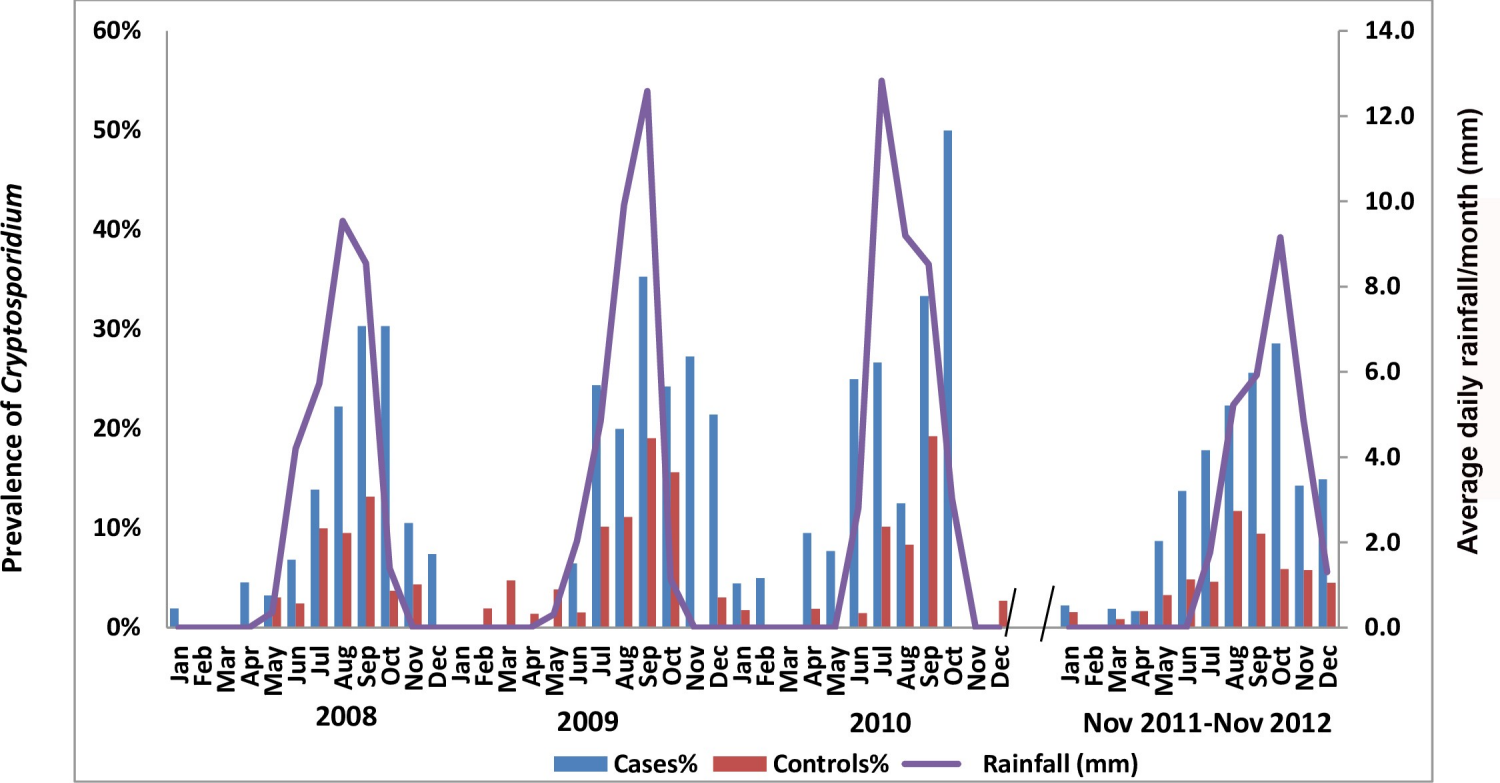
Prevalence of *Cryptosporidium* in diarrhea cases and controls in relation with months, years (2008–2012) and rainfall.

*Cryptosporidium* prevalence was higher in cases than in controls in both the dry and wet seasons (Table 1). Furthermore, relative to dry season, *Cryptosporidium* is much more prevalent in both cases and controls during the wet and rainy season (May-October). The overall prevalence of *Cryptosporidium* in both cases and controls usually started to rise in May, with a peak between July and October (Fig 2). *Cryptosporidium* diarrhea cases peaked in September (28.3% of all cases) and October (28.2% of cases); prevalence was low between January and April (range, 0.64%-2.8%).

### Prevalence of *Cryptosporidium* species

*Cryptosporidium* results from the TaqMan assay were available for 1506 stool samples (759 cases and 747 controls); 280 (18.6%) of these were positive for *Cryptosporidium* by TaqMan PCR, 121 (8.0%) were positive by EIA, and 104 (6.9%) were positive by both assays. The TaqMan assay was used to identify samples positive for *C*. *hominius* or *C*. *parvum*. Of the 280 positives by Taqman, 119 (42.5%) were positive for *C*. *hominis*, and 20 (7.1%) were positive for *C*. *parvum* and 5 (1.8%) were positive for both. Among 144 samples with both species identified, only *C*. *hominis* was in 119 (82.6%), only *C*. p*arvum* in 20 (13.9%), and both *C*. *hominis* and *C*. p*arvum* was in 5 (3.5%). *C*. *hominis* was more frequently positive in cases than in controls (78/753, 10.4% vs. 46/741, 6.2%; p = 0.004). The frequencies of *C*. *parvum* were similar in cases and controls (12/746, 1.6% vs. 13/739, 1.8%; p = 0.82).

### Risk factors for *Cryptosporidium*-positive diarrhea

Analysis of risk factors included 231 *Cryptosporidium*-positive diarrhea cases and 349 matched controls; 36 (10.3%) of the controls were positive for *Cryptosporidium* infection (Table 2). One hundred sixty-seven (72.3%) of the cases had MSD and 64 (27.7%) had LSD. Since controls were matched to cases by age and sex, no analysis of these variables was done. Results of univariable conditional regression analyses are summarized in Table 2 for socioeconomic characteristics, animals living in the compound, water source and hygiene, breast feeding status, nutritional status, and the presence of other putative pathogens.

**Table 2:** Association of risk factors with *Cryptosporidium* diarrhea: univariable conditional logistic regression analysis.

Exposure	Cryptosporidium cases (N = 231) n (%)	Matched controls (N = 349) n (%)	Odds Ratio	95% CI	P^a^
**Demographic characteristics**					
Sex: Female^b^	100 (43.3)	153 (43.8)	-	-	—
Age stratum (months)^b^					
0–11	113 (48.9)	167 (47.9)	-	-	—
12–23	99 (42.9)	147 (42.1)	-	-	—
24–59	19 (8.2)	35 (10.0)	-	-	—
**Socioeconomic characteristics**					
Primary caretaker					
Mother	227 (98.3)	347 (99.4)	0.36	0.06–2.0	0.24
Completed primary schooling	17/229 (7.4)	22/347 (6.3)	1.1	0.51–2.2	0.89
Respondent: Mother	227 (98.3)	348 (99.7)	0.22	0.03–1.7	0.15
Mother lives in household	229 (99.1)	347 (99.4)	0.84	0.12–6.1	0.87
Father lives in household	172 (74.5)	257 (73.6)	1.0	0.68–1.6	0.87
Housing: non-finished floor	27 (11.7)	40 (11.5)	0.9	0.58–1.9	0.90
Mean wealth index^c^ (SD)	0.02 (0.99)	0.05 (0.94)	1.0	0.80–1.3	0.99
Number of household members in last 6 months (in units of 5)					
Mean (SD) Median (min, max)	6.0 (3.9) 5.0 (0.6, 22.4)	7.7 (4.8) 6.8 (0.6, 23.6)	0.88	0.84–0.93	<0.001
>20 people	142 (61.5)	257 (73.6)	0.52	0.35–0.77	0.001
**Animals living in the compound**					
Goat	175 (75.8)	280 (80.2)	0.77	0.50–1.2	0.24
Sheep	172 (74.5)	271 (77.7)	0.82	0.55–1.2	0.35
Dog	60 (26.0)	76 (21.8)	1.4	0.85–2.2	0.19
Cat	70 (30.3)	52 (14.9)	2.4	1.5–3.6	<0.001
Cow	86 (37.2)	73 (20.9)	2.9	1.8–4.6	<0.001
Horse or Donkey	151 (65.4)	22 6 (64.8)	0.91	0.60–1.4	0.64
Rodents	180 (77.9)	244 (69.9)	1.8	1.2–2.7	0.008
Fowl (Chicken, duck or other birds)	193 (83.5)	311 (89.1)	0.54	0.31–0.92	0.023
**Water source and hygiene**					
Improved drinking water^d^ used in last two weeks	127 (55.0)	194 (55.6)	0.98	0.66–1.5	0.91
Child drank stored water at home in last two weeks	205 (88.7)	254 (72.9)	5.5	2.7–10.9	<0.001
Usually treat (filter) drinking water through cloth^e^	67 (29.0)	77 (22.1)	1.6	1.0–2.7	0.043
Usually dispose of child’s feces in yard, bush, open spaces	13 (5.6)	10 (2.9)	2.2	0.87–5.6	0.087
The Household most commonly uses an improved toilet compared to a traditional pit toilet	7 (3.0)	4 (1.2)	2.6	0.75–9.2	0.13
**Current breast feeding status by age strata**					
Partial or exclusive breast feeding	180 (77.9)	270 (77.4)	0.87	0.46–1.6	0.67
0–11 months	112/113 (99.1)	165/167 (98.8)	1.1	0.10–11.4	0.96
12–23 months	66/99 (66.7)	104/147 (70.7)	0.75	0.38–1.5	0.42
24–59 months	2/19 (10.5)	1/349 (2.9)	2.7	0.25 28.9	0.41
**Nutritional status**					
Stunted (height for age Z-score < -2)	60 (26.0)	89/348 (25.6)	1.1	0.71–1.6	0.77
**Presence of other pathogens**^**f**^							
Rotavirus	8 (3.5)	7 (2.0)	2.3	0.66–8.3	0.19
Norovirus GII	13 (5.6)	20 (5.7)	0.94	0.44–2.0	0.87
Adenovirus 40/41	1 (0.4)	1 (0.3)	1.4	0.08–23.6	0.81
*Shigella*	5 (2.2)	3 (0.9)	2.9	0.68–12.6	0.15
ETEC-ST	24 (10.4)	31 (8.9)	1.1	0.60–2.0	0.79
*Giardia*	28 (12.1)	99 (28.4)	0.33	0.20–0.55	<0.001
*Entamoeba histolytica*	0(0.0)	1 (0.29)	-	-	-
*Ascaris lumbricoides*	5/117 (4.3)	9/163 (5.5)	0.74	0.25–2.2	0.60

^a^ From conditional logistic regression using the Firth penalized likelihood.

^b^Conditional logistic regression analysis was not done for age and sex, since controls were matched to cases by these variables.

SD = Standard deviation

^c^ Wealth index was calculated by factor analysis, using electricity in the household and ownership of television, radio, phone, bike, car, boat, refrigerator and finished floor (y/n) and number of sleeping rooms in the household [29].

^d^Improved drinking water: The main source of drinking water in the last 2 weeks for household members is either piped into house or yard, public tap, deep or shallow tube well, covered well or bore hole, takes <15 mins to collect, and is available daily.

^e^Compared to no usual treatment of drinking water or other methods of treatment

^f^Pathogens most strongly associated with moderate-to-severe diarrhea, as well as parasitic infections, are included in the list of other pathogens.

In Table 2, the median numbers of household members for *Cryptosporidium*-positive diarrhea cases and their matched controls were 25 (range, 3–112) and 34 (range, 3–118), respectively. The mean number of household members and the proportion households with more than 20 members were significantly higher in controls than in cases.

Children with *Cryptosporidium*-positive diarrhea were significantly more likely than their matched controls to live in a compound with a cat, cow or rodents. Fowl (chicken, duck or other birds) living in the compound was more common in controls than cases.

*Cryptosporidium*-positive diarrhea was significantly associated with drinking stored water at home in the last two weeks and drinking water usually filtered through a cloth. The proportions of cases and controls currently being breast fed, as well as the proportions of cases and controls with stunting, were similar and not significantly different.

The proportion of *Cryptosporidium*-positive diarrhea cases co-infected with *Giardia*, the most common protozoal co-infection, was significantly lower than the proportion in their matched controls. No other co-infection evaluated was significantly associated with *Cryptosporidium*-positive diarrhea.

Table 3 summarizes the results of multivariable conditional logistic regression modeling for associations with *Cryptosporidium*-positive diarrhea. Inclusion of interactions with an MSD/LSD indicator variable allowed development of separate models for *Cryptosporidium*-positive MSD and *Cryptosporidium*-positive LSD. All factors in the table were significantly associated (95% confidence interval for odds ratio did not include 1) for either MSD, LSD, or both. There was an increased risk of *Cryptosporidium*-positive MSD and/or LSD for certain animals (cow, cat, rodents) in the household, as well as the child drinking stored drinking water at home in the last two weeks. There were significant differences (p < 0.05 for interaction term) between MSD and LSD for odds ratios for a cow or cat living in the compound and a suggestion of a difference (p = 0.069) for rodents in the compound. There was no evidence of a difference between MSD and LSD in the association with number of people living in the household (in units of 5 individuals), child consuming stored drinking water at home in the last two weeks, fowl living in the compound or mixed infection with *Giardia*.

**Table 3:** Association of risk factors with *Cryptosporidium*-positive MSD and LSD separately: Multivariable conditional logistic regression analysis.

	*Cryptosporidium*-positive MSD		*Cryptosporidium*- positive LSD		
Exposures	OR^1^	95% CI^2^	OR^1^	95% CI^2^	P*
Number of regular household members in last 6 weeks (in units of 5)	0.87	0.81–0.93	0.82	0.71–0.94	0.41
Animals living in the compound					
Cow	2.1	1.1–3.9	16.7	3.2–87.5	0.022
Cat	3.5	1.8–6.8	0.43	0.14–1.3	0.001
Rodents	1.5	0.82–2.7	5.4	1.5–18.9	0.069
Fowl (chicken, duck or other birds)	0.38	0.17–0.82	0.19	0.04–0.87	0.43
Child drank stored drinking water at home in last 2 weeks	4.6	2.0–10.2	2.9	0.24–34.0	0.73
Mixed infection with Giardia	0.29	0.14–0.60	0.18	0.05–0.67	0.54

^1^ OR: odds ratio

^2^ CI: confidence interval

* p-value for testing difference between ORs for MSD and LSD (i.e., for interaction with MSD/LSD indicator variable).

## Discussion

Our findings in this comprehensive population-based epidemiological study of endemic *Cryptosporidium* infection in young Gambian children show a striking association of *Cryptosporidium*-positive diarrhea with age, with nearly all cases (86%) occurring in children 6–23 months of age. A similar trend with a lower overall prevalence of *Cryptosporidium*-positive diarrhea, was observed in a study conducted in urban Gambia during 1991–1992 [18]. Other studies in sub-Saharan Africa have shown roughly similar prevalence but may lack the representativeness of long-term population-based studies with improved diagnostic techniques [21]. The low prevalence in infants 0–5 months of age may be explained by exclusive breast feeding [31] or transfer of immunity from mother to child. The high prevalence at 6–23 months of age may be due to exposure to contaminated food and/or water in the weaning period. Stenberg *et al*. demonstrated in a sero-prevalence study in Guatemala that the prevalence of antibody to *Cryptosporidium parvum* increased at older ages compared to those aged 6–12 months [32]. Furthermore, a study in healthy adult volunteers showed that higher anti-*Cryptosporidium* IgG antibody levels were associated with a reduced chance of infection and illness when challenged with low *Cryptosporidium* oocyst doses [33]. Thus, the low prevalence of *Cryptosporidium* in children 24–59 months of age may relate to the development of immunity following earlier infection. A decreased risk of infection in older children due to the development of partial immunity from earlier exposure would suggest that a vaccine may protect against *Cryptosporidium* infection.

The population HIV prevalence is low in our study area (<2%) [22]. Our findings show that *Cryptosporidium* is an important infection in young children even in a population with low HIV prevalence.

*C*. *hominis* is the predominant among identified species of *C*. *hominis* and *C*. *Parvum* (82.6% vs. 13.9%) in the Gambia. This finding is consistent with results from other studies in Kenya, Malawi, Uganda, Bangladesh and India [5, 11, 16, 34, 35]. It suggests that the primary mode of transmission in The Gambia is anthroponotic transmission, although there may also be zoonotic transmission. Either may occur through contact with contaminated drinking water [19]. Accordingly, we found consumption of stored drinking water to be associated with increased risk of *Cryptosporidium* diarrhea.

In our study, 81% of *Cryptosporidium*-positive diarrhea cases and 79% of *Cryptosporidium*-positive controls occurred during the rainy season. Earlier studies in The Gambia [18], Madagascar [36], Guinea-Bissau [37], Brazil [38, 39] and India [40] found associations between *Cryptosporidium* diarrhea and rainy season. The association of rainfall and *Cryptosporidium* infection could be due to domestic use of surface water, contamination of unprotected wells, and poor hygiene practices. In the rainy season, surface water may be more often contaminated with human and animal feces, so that children playing in the contaminated surface or stagnant water could facilitate the transmission of *Cryptosporidium*. However, *Cryptosporidium* infection has also been observed predominantly in the dry season in Kenya and Guatemala, when drinking water is limited [34, 41].

In our study area, drinking water is usually stored in wide-mouth containers. Household members and children use their hands to dip a cup into the water storage container to obtain drinking water, which may further contaminate the water. The chance of contamination may be increased with prolonged storage and handling if the storage container is not frequently cleaned. Further exploration of storage practices, sanitation and hygiene measures for collection of water and removing water from the container, as well as further laboratory analysis of potable water from the source to storage and point of consumption may all help to establish the source of infection.

Contrary to the findings of an association of household overcrowding with increased risk of cryptosporidiosis, our study found the opposite. The reasons for this negative association are not clear. In an urban area, close proximity of houses, overcrowding, close personal contract and lack of sanitary facilities may contribute to the spread of *C*. *hominis* infection [42]. Perhaps in a rural setting, the greater physical space available per person leads to less close contact between children in the household.

We found that the presence in the compound of domestic animals (cattle and cats) and the presence of rodents in the compound were potential risk factors for *Cryptosporidium* diarrhea. Association of *Cryptosporidium* diarrhea with the presence of animals was also found in Guinea Bissau and Guatemala [41, 43]. We believe that our study is the first to suggest an association between rodents living in a household and the presence of *Cryptosporidium* diarrhea. Human carriage of *Cryptosporidium muris*, a predominantly rodent pathogen, and rodents have been identified as reservoirs of *C*. *parvum* and *C*. *hominis* [44, 45].

The presence of *Giardia* was inversely associated with *Cryptosporidium* diarrhea. Similarly, a longitudinal analysis in another study showed no evidence of an association between *Giardia* infection and an increased risk of diarrhea [46]. In a systematic review, giardiasis was associated with decreased risk of acute diarrhea in children in developing countries [47]; however, the same review found that *Giardia* infection was positively associated with persistent diarrhea and suggested that initial *Giardia* infections early in infancy may be positively associated with diarrhea. *Giardia* may secrete mucins and glycoproteins in the intestinal mucosal layer, which may protect against attachment of other pathogens including *Cryptosporidium*, and such a mechanism may protect against *Cryptosporidium*-positive diarrhea [48].

A limitation of the study is that only about 68% of *Cryptosporidium*-positive diarrhea cases can be attributed to *Cryptosporidium*. Thus, some of the risk factor associations that we found could be at least partially due to factors other than the presence of *Cryptosporidium*. However, it is clear both from the present study and other studies that infants and young children in developing countries with *Cryptosporidium*-positive diarrhea are at risk of negative health consequences and that reducing the level of *Cryptosporidium* infection is an important public health concern in these countries.

## Conclusion

Our study establishes that *Cryptosporidium* is an important cause of childhood diarrhea in The Gambia. Data from the ongoing rotavirus vaccine impact study will help us understand the burden of *Cryptosporidium* infection in Africa after introduction of rotavirus vaccine in routine immunization programs. The drinking stored water and animals living in the household are associated with *Cryptosporidium-*positive diarrhea. The predominance of *C*. *hominis* suggests anthroponotic transmission of *Cryptosporidium* infection. Associations of *Cryptosporidium-*positive diarrhea with drinking of stored water and animals living in the household suggest there may also be zoonotic transmission. Thus, general improved hygienic practices to store drinking water may reduce transmission of *Cryptosporidium*. The role of animals in the transmission of *Cryptosporidium*, the methods of drinking water storage, and sanitation and hygiene measures used for taking water from the water storage containers merit further study.
